# Fungal Diseases in the 21st Century: The Near and Far Horizons

**DOI:** 10.20411/pai.v3i2.249

**Published:** 2018-09-25

**Authors:** Arturo Casadevall

**Affiliations:** Department of Molecular Microbiology and Immunology, The Johns Hopkins School of Public Health, Baltimore, Maryland

**Keywords:** Fungal Diseases, mycology, immunotherapies, drug resistance, diagnostics, global warming

## Abstract

Fungal diseases became a major medical problem in the second half of the 20th century when advances in modern medicine together with the HIV epidemic resulted in large numbers of individuals with impaired immunity. Fungal diseases are difficult to manage because they tend to be chronic, hard to diagnose, and difficult to eradicate with antifungal drugs. This essay considers the future of medical mycology in the 21st century, extrapolating from current trends. In the near horizon, the prevalence of fungal diseases is likely to increase, as there will be more hosts with impaired immunity and drug resistance will inevitably increase after selection by antifungal drug use. We can expect progress in the development of new drugs, diagnostics, vaccines, and immunotherapies. In the far horizon, humanity may face new fungal diseases in association with climate change. Some current associations between chronic diseases and fungal infections could lead to the establishment of fungi as causative agents, which will greatly enhance their medical importance. All trends suggest that the importance of fungal diseases will increase in the 21st century, and enhanced human preparedness for this scourge will require more research investment in this group of infectious diseases.

To many observers, medical mycology is the new kid on the block relative to bacteriology, virology, and parasitology. This impression has arisen because fungal diseases became a major medical problem only in the second half of the 20th century with the confluence of the advances in medicine and the initiation of the HIV epidemic, which resulted in large numbers of human hosts with impaired immunity. However, like many perceptions and impressions, the historical facts imply otherwise. Medical mycology can claim that it was the first discipline to establish that a microbe can cause human disease when David Gruby showed that favus was a fungal disease in mid-19th century France. Earlier in that century, Agostino Bassi had demonstrated that *Beauveria bassiana* caused disease in silkworms providing the first firm observation for the germ theory of disease. Hence, medical mycology as a branch of the larger discipline of mycology has a long and distinguished record of medical contributions dating back over a century. Today medical mycology is thriving scientifically as the study of fungal pathogens reveals new fundamental problems different from those posed by the other classes of pathogenic microbes. In this essay, I will try to envisage the near and far horizons keeping in mind the adage and warning that “prediction is difficult, especially about the future,” a saying that has been variously attributed to Nostradamus, Niels Bohr, and most recently, Yogi Berra.

In pondering what to include in this essay I imagined what I would have written if invited to produce a similar article in 1975. At the time, I could not have imagined the AIDS epidemic when a previously unknown virus that was already spreading through the world would infect large numbers of individuals, devastate their immune systems and leave them at high risk for fungal diseases. By the early 1990s, that epidemic was to make cryptococcal meningitis the most common cause of culture positive meningitis in New York City [1]; a fact that would have been unfathomable two decades earlier. Hence, the thoughts in this essay should be taken with a grain of salt, meaning with caution and skepticism. If prediction is difficult and unreliable, why bother? This is a fair question, and I can think of various responses. In imagining what is ahead, we reveal how we think current trends will develop, and the prescience, or lack thereof, can inform future readers of our concerns. Pondering the future is also the only mechanism available for anticipating needs and trends. Hence, I do think the exercise is valuable despite its epistemological limitations.

In this essay, I will focus primarily on human fungal diseases while referring frequently to the problem of mycotic infections throughout the biota. We are living at a time when mycotic diseases are devastating entire ecosystems having emerged as major pathogens of amphibians, bats, turtles, snakes, and food producing plants [2]. One of the most remarkable aspects of mammalian physiology is its tremendous resistance to invasive fungal diseases. While fungi are major pathogens of insects, amphibians, reptiles, and plants, there are relatively few fungal diseases of mammals, which we have attributed to the combination of endothermy and adaptive immunity [3]. Mammalian endothermy creates a thermally restricted space for most fungal species, which when combined with innate and adaptive immunity, makes invasive fungal diseases rare among immunologically intact humans [4]. In fact, the human core temperature appears to represent a maximal tradeoff between that which excludes most fungal species while maintaining manageable energetic costs, suggesting an evolutionary connection between endothermy and protection against fungi [5]. The connection between the high temperatures of mammals their resistance to fungal disease could have contributed to the great mammalian radiation following the cataclysm at the end of the Cretaceous period, where their high temperatures would have made them resistant to fungal pathogens [3, 6]. The rarity of fungal diseases in humans with apparently normal immunity suggests why their description was later than for many bacterial diseases. For example, cryptococcosis, histoplasmosis, coccidiomycosis, and paracococcidiodomycosis were each described between 1890 and 1910, usually as individual case reports that came to medical attention because of the rarity of these conditions.

## THE CURRENT SITUATION

The medical, veterinary, and ecological importance of fungal diseases has increased dramatically in the past half century. The current burden of mycotic diseases in humans exceeds several million cases worldwide [7, 8]. The causes for the current situation are complex, but they mostly reflect a confluence of human activities that have resulted in medical progress, travel, and commerce combined with the cataclysm of the HIV epidemic. In the medical arena, fungal diseases have followed the use of antibiotics that disrupt the normal microbiome, the treatment of cancers with drugs that impair immunity, the use of immunosuppressive agents to treat autoimmune diseases, and such invasive procedures as the placement of intravascular catheters and surgery. In the broader arena of biology, fungal diseases are currently devastating entire ecosystems [2, 7].

Fungal diseases differ from most bacterial diseases in that they tend to be chronic and kill the host slowly. Fungal diseases are also more recalcitrant to therapy such that most invasive mycoses require treatment courses lasting months or longer. In contrast to bacterial and viral diseases, invasive human fungal infections are rarely communicable, and this has led to reduced interest by public health authorities in surveillance, so that we have relatively little information on the incidence and prevalence of mycoses. Fungal epidemics, when they occur, tend to reflect one of three events: 1) increased prevalence of hosts with compromised immunity susceptible to diseases (eg cryptococcosis epidemics in patients with advanced HIV infection [9]); 2) exposures to a large inoculum, such as outbreaks of histoplasmosis following tree cuttings in endemic areas [10]; and 3) iatrogenic causes such as the outbreak of *Exserohilum rostratum* fungal meningitis following contaminated steroid solutions [11]. In contrast, some animal mycoses like the white-nose syndrome in bats are communicable among community members and the lack of contagiousness in human diseases tends to reflect specific characteristics of those organisms rather than a property of fungi.

The therapy for most invasive fungal diseases remains unsatisfactory given their high morbidity and mortality despite the best available antifungal treatment. For example, fungal diseases such as aspergillosis and cryptococcosis have high mortality even when treated with appropriate therapy and are often incurable in hosts with impaired immunity. The chronicity of some fungal diseases requires prolonged therapy, which in turn increases the risk of development of antifungal resistance. Hence, the current situation is one where fungal diseases are increasingly prevalent in both human and animals with unsatisfactory treatment options for the former and few or none for the latter. These diseases are increasingly associated with antifungal drug resistance. New pathogenic fungi have appeared, such as *Candida auris*, which manifest a high degree of resistance to existing antifungal agents and have caused outbreaks in intensive care units.

Despite devastation caused by fungal diseases in susceptible humans and in various ecosystems, the fungal kingdom tends to be ignored relative to bacteria, viruses, and parasites. The journal *Nature Microbiology* recently pointed this out with an editorial titled “Stop neglecting fungi” [12]. However, there has been some recent progress in recognition of mycotic diseases, especially in the United Kingdom [13].

## THE NEAR HORIZON

### New Populations at Risk for Fungal Diseases

For the near horizon, it is likely that the current trends of an increasing prevalence of fungal diseases will continue. Considering that modern medicine is in the midst of a revolution in antibody-based immunotherapies that target specific immune molecules and cells, we can anticipate an increasing number of individuals at risk for invasive fungal diseases [14]. For example, the introduction of revolutionary tumor necrosis blockage therapy for rheumatologic diseases created a new type of host susceptible to fungal diseases [15].

### Vaccines and Immunotherapies

We are likely to see continued progress in the development of vaccines and immunotherapies for fungal diseases despite considerable hurdles in clinical development of such entities [16]. Currently there are no vaccines or immune therapies for fungal diseases, but many are in development. A recent success was the demonstration that a vaccine to prevent recurrence of vaginal candidiasis was safe and effective [17]. It is noteworthy that this vaccine did not prevent infection but rather functioned as a therapeutic vaccine to prevent disease in hosts that were chronically colonized with *Candida albicans* [18]. In the area of immunotherapies, some of the most promising leads are monoclonal antibodies (mAb), of which several have been reported to protect against fungal infections in animal models (reviewed in [19]), and one mAb to *Cryptococcus neoformans* was taken to clinical Phase 1 testing [20]. A mAb to poly-N-acetyl glucosamine prevented fungal keratitis in mice, raising the possibility of developing an immunotherapy against this serious ocular infection [21]. Also of particular interest are antibodies that target epitopes common to bacterial and fungal pathogens as this increases the potential market size of the product, which could be an incentive for their development. In this regard, a mAb targeting sialylated oligosaccharides of Group B streptococcus protected against experimental aspergillosis in mice [22]. Given that the majority of invasive fungal diseases occur in individuals with impaired immunity, attempts to reduce host susceptibility by manipulating the immune system with vaccines and immunotherapy make sense, which is likely to stimulate their continued development.

### Diagnostics

One of the axioms in medical mycology is that diagnosis of invasive fungal infections is difficult so that many are definitively diagnosed only postmortem [23]. For decades, the only reliable serological diagnostic test was the cryptococcal antigen detection assay developed in the 1960s. This assay has evolved into a point-of-care diagnostic test using the lateral antigen flow technique that allows rapid diagnosis of cryptococcal meningitis in underserved areas [24]. However, recent decades have witnessed steady progress in the detection of fungal products in blood such as galactomannan and 1, 3 β-D-glucan for diagnosing invasive aspergillosis [23]. Mass spectrometry is a promising technology for diagnosing invasive fungal infections because it can detect fungal metabolites in serum [25]. Similarly, nucleic acid detection methods for fungal pathogens can perform well in certain circumstances and provide yet another strategy for diagnosing mycoses [26, 27]. The ongoing revolution in imaging could also produce significant improvements in diagnosis as shown by detection of experimental aspergillosis in mice using a combination of positron emission tomography and MRI [28]. The simultaneous development of several technologically independent diagnostic techniques suggests that we can expect continued and steady progress in the diagnosis of fungal diseases premortem in the near term.

### Antifungal Drug Resistance

Like the situation with antibacterial drugs, the use of antifungal agents in clinical, veterinary, and agricultural settings is associated with increasing drug resistance in clinically relevant fungi [29]. In addition to increasing resistance among established fungal pathogens, recent years have seen the appearance of the highly resistant *C. auris* as a major cause of nosocomial fungal infection worldwide [30]. The emergence of antifungal drug resistance has usually been associated with situations requiring chronic antifungal use in individuals with persistent infection where the drug produces a selective pressure for less drug-susceptible variants. Although resistance has been less of a concern for fungal diseases acquired from the environment, there is some evidence that the use of azoles in agriculture is resulting in more clinical isolates with lower susceptibility to these drugs [31, 32]. Given the trend to treat increasing numbers of fungal diseases with antifungal drugs, the use of azoles to control plant fungal diseases in agriculture and the resulting selection pressure for resistance suggests a high likelihood that the problem of antifungal drug resistance will worsen in the near future. The mechanisms relating to altered patterns of gene expression that can reduce bacterial drug susceptibility and suggestions that these processes could also apply to fungal pathogens should be investigated in the context of treatment of mycotic diseases.

### New Antifungal Agents

For decades the primary antifungal formulary reflected a paucity of drug classes and was limited to polyenes, azoles, and most recently echinocandins. Invasive fungal diseases were invariably fatal until the polyene amphotericin B was introduced into clinical use in the late 1950s, a full two decades after the availability of the first effective antibacterial drugs. In the six decades since amphotericin B was introduced it has retained its role as the premier antifungal therapy despite high toxicity, but beginning in the 1990s it was replaced as first line therapy for some fungal disease by azoles and echinocandins. However, the mortality and morbidity of fungal diseases has remained stubbornly high despite antifungal therapy, which probably reflects the fact that these diseases occur in individuals with impaired immunity, hosts where antifungal therapy is not enough to clear the infection. In fact, John Perfect has convincingly argued that the effectiveness of current antifungal regimens has “plateaued” [33]. There is hope that as new classes of antifungal agents are developed, the prognosis of individuals with invasive fungal diseases will improve, possibly by the use of combinations that can effectively kill the fungal burden in tissue even in settings of impaired immunity. Interesting areas of new antifungal drug discovery include the identification of antifungal activity in drugs designed for other purposes. Sertroline, an antidepressant, was active against *C. neoformans* in a murine model of cryptococcosis [34], although subsequent clinical testing did not find a benefit in combination with standard antifungal therapy [35]. Similarly, there are several compounds among molecules with activity against malaria that are candidates for development as antifungal agents [36, 37]. In addition, there are numerous antifungal drugs in the developmental pipeline, and some of these are likely to become available for clinical use in the near future [33].

### Mycobiome

During the microbiome revolution, fungal components of the microbiota have received very little attention compared to bacterial communities [38]. Fortunately, this is beginning to change with the publication of several major studies describing the human mycobiome in different body sites; a development pioneered by Dr. Mahmoud Ghannoum and colleagues [39]. There is now great appreciation that the mycobiome is a critical component of the microbiome and that fungal-bacterial interactions have major effects on human health [40–43]. Hence, we can anticipate that the mycobiome will continue to be studied with the goal of understanding how the prevalence of certain fungal species impacts health and disease. Once the role of fungi in the microbiota-host interaction are better understood, future efforts may shift towards identifying how purposeful modification of the mycobiome can be used to prevent and treat certain diseases.

### New Disease Associations

In recent years there have been several reports associating fungal diseases with medical conditions that are generally considered unrelated to mycoses. Fungal sensitization has been associated with severe childhood asthma [44]. Another study showed that 50% of adult asthmatics had fungal sensitization, which suggests that reactions to fungal antigens could be contributing to disease in millions of individuals [45]. Pathogenic fungi are often detected in the lungs of individuals with cystic fibrosis [46]. Whether these fungi are merely colonizing the cystic fibrosis secretions or contributing to disease is unknown. Recently, there has been the provocative report that fungi can be detected in Alzheimer's lesions by DNA sequencing [47, 48]. Finally, Pneumocystis infection has been associated with infant death syndrome [49]. At the current time, none of the associations between these diseases and fungal infection is known to be causative. However, if future research reveals a causative association between fungal infections and any of these severe diseases, that would greatly elevate the medical importance of fungi.

## THE FAR HORIZON

### Global Warming and New Fungal Diseases

**Figure 1.**
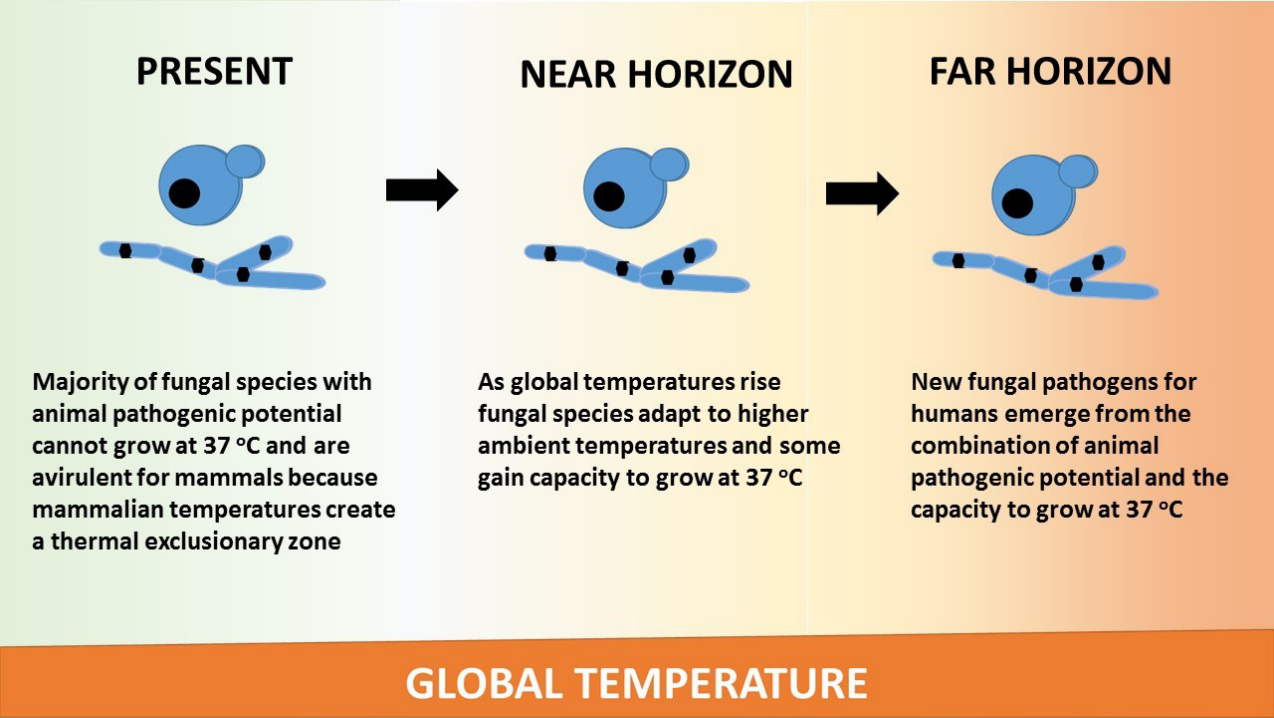
**Global warming is hypothesized to result in the emergence of new fungal pathogens.** The mechanism for this effect posits that fungi will adapt to higher temperatures by increasing their thermal tolerance. Among those species that adapt are some with pathogenic potential that are currently nonpathogenic for mammals because they cannot thrive at mammalian temperatures. However, adaptation to higher temperatures will defeat the mammalian thermal exclusionary zone, and consequently, we may encounter new fungal pathogens as the planet warms.

Climate predictions for the 21st century envision progressive warming. As the climate warms, the gradient between mammalian and ambient temperatures narrows, and this combined with the ability of fungi to adapt to higher temperatures raises the possibility for new fungal diseases [50, 51] (Figure 1). This hypothesis posits that that there are large numbers of fungal species with the potential to be virulent for mammals that are currently nonpathogenic by virtue of the fact that they cannot replicate at mammalian temperatures [52]. The thermal mismatch hypothesis argues that as climate gets warmer, temperatures are shifted away from the range optimal for hosts to resist certain pathogens [52]. Testing of this hypothesis by analyzing *Batrachochytrium dendrobatidis* susceptibility for various hosts provided evidence that as climate warmed host susceptibility increased. Another potential effect of global warming will be an increased prevalence of thermophilic fungi that produce mycotoxins thus posing another threat to human health through their secondary metabolites [53]. Hence, new fungal diseases are likely to threaten humans and their biosphere in the far horizon.

### Immunotherapy

Because the majority of invasive human fungal diseases occur in individuals with impaired immunity, it is likely that further improvements in therapy will require therapies that reverse the immune defect. We already have an example of this approach by the observation that individuals with advanced HIV infection who are at risk for such diseases as cryptococcosis and histoplasmosis regain their resistance to disease when antiretroviral therapy improves immune function. We have also witnessed paradoxical worsening of individuals recovering from fungal diseases when rebounding host immune function from antiretroviral therapy triggers inflammatory damage (eg, immune reconstitution inflammatory syndrome or IRIS [54]). To avoid this outcome will require much greater knowledge of immunology and the ability to manipulate host responses. The damage response framework of microbial pathogenesis posits that host damage leading to disease occurs at both horns of the parabola generated when damage is considered as a function of the immune response [55]. For example, *C. neoformans* infection causes disease when sufficient damage is mediated by the infection to affect homeostasis, and this damage can occur in the setting of both weak and strong immune responses [56, 57]. One of the hurdles in using immunotherapy is that currently physicians do not know whether the damage being incurred by the host is a result of inappropriately weak or strong immune responses. Taking the long view one can anticipate a time when physicians can determine the source and type of host damage in real time and administer therapy that will precisely tune the system to clear the microbe and lessen host damage.

### NonInvasive Diagnostics

Invasive fungal diseases tend to kill the host slowly and are often associated with a large microbial burden in tissue. Given that fungal metabolism is very different from mammalian metabolism, mycotic diseases result in the accumulation in tissue of high concentrations of fungal products that can be detected by nuclear magnetic resonance (NMR). Diagnosis of cryptococcal meningitis by NMR analysis of cerebrospinal fluid is already possible [58]. Similar success was reported in the identification of Candida in blood [59]. The fungal burden in cryptococcal infection is so large that fungal products can be detected in tissue by nuclear magnetic resonance [60]. One can imagine a future where it may be possible to diagnose mycotic diseases by magnetic resonance imaging if sufficiently powerful machines are developed. Fungi also produce many unique volatiles that can be detected in breath [61]. Hence, the future is likely to see the development of rapid noninvasive diagnostics based on new technologies.

## CLOSING THOUGHTS

The situation with invasive fungal diseases is currently unsatisfactory as they remain difficult to diagnose and treat. In future years, the problem posed by invasive fungal infections is likely to grow, and humanity may yet confront new threats from the fungal kingdom as currently non-threatening but potentially pathogenic species adapt to warmer temperatures and thus defeat the thermal restriction zone conferred by mammalian endothermy. Despite the gravity of the threat, we can anticipate considerable progress in our capacity to prevent, diagnose, and treat fungal diseases in both the short- and long-term. However, humanity's investment in research into fungal diseases remains very low given the potential threat, especially when compared to the investment/threat ratio for other infectious diseases [8]. One of the best documented examples of underfunding for mycotic diseases is cryptococcosis. This diseases is currently responsible for 15%-20% of deaths in individuals with AIDS, and yet in 2015 it received 1% of the funding allocated to tuberculosis [62] despite the fact that deaths from cryptococcosis exceed those from tuberculosis in Africa [9]. Remedying this situation requires more advocacy by the infectious disease community as well as more studies documenting the global burden of fungal diseases and their impact on human health.
